# Associations Between Oral Health Status, Perceived Stress, and Neuropsychiatric Symptoms Among Community Individuals With Alzheimer's Disease: A Mediation Analysis

**DOI:** 10.3389/fnagi.2021.801209

**Published:** 2022-01-10

**Authors:** Bing Yang, Binbin Tao, Qianyu Yin, Zhaowu Chai, Ling Xu, Qinghua Zhao, Jun Wang

**Affiliations:** ^1^Department of Nursing, Stomatological Hospital of Chongqing Medical University, Chongqing, China; ^2^Department of Nursing, The First Affiliated Hospital of Chongqing Medical University, Chongqing, China; ^3^Community Health Center of Daxigou, Chongqing, China

**Keywords:** Alzheimer's disease, oral health, dementia, perceived stress, mild cognitive impairment, subjective cognitive decline, stress process model

## Abstract

Community individuals with Alzheimer's disease (AD) experience oral disease alongside neuropsychiatric symptoms (NPS) with disease progression. Despite growing evidence for the link between oral health and cognitive status, few studies have investigated the associations between oral health and NPS, especially based on individuals' experience of AD. The primary aim of this study was to examine (a) the difference in oral health-related stressors among individuals with AD, mild cognitive impairment (MCI), and subjective cognitive decline (SCD); and (b) the associations of these stressors with NPS under the framework of the stress process model (SPM). A cross-sectional study was conducted among individuals diagnosed with AD (*n* = 35), MCI (*n* = 36) or SCD (*n* = 35), matched for age, sex education, and body mass index (BMI). Multiple regression and mediation model analyses were performed to explore predictors and their relationships with NPS based on the SPM. Data collection comprised four sections: (a) individual context; (b) oral health-related stressors, including dental caries, periodontal status, oral hygiene, the geriatric oral health assessment index (GOHAI), oral salivary microbiota, pro-inflammatory cytokines, and oral health behavior; (c) subjective stressors (i.e., perceived stress [PS]); and (d) NPS. Decayed, missing, and filled teeth (DMFT), missing teeth (MT), loss of attachment (LoA), plaque index (PLI), PS, oral health behavior, GOHAI, pro-inflammatory cytokines, and salivary bacterial composition were significantly different among the three groups; these parameters were poorer in the AD group than SCD and/or MCI group. LoA, PLI, PS, and pain or discomfort in the GOHAI were directly associated with NPS. PLI, LoA, and psychosocial function in the GOHAI indirectly affected NPS, and this relationship was mediated by PS. Individuals with AD reported greater oral health-related stressors. This study identifies direct and indirect associations linking oral health-related stressors and PS with NPS in individuals with AD. Our findings suggest that targeted dental care and oral-related stressor control may be valuable for managing NPS.

## Introduction

Alzheimer's disease (AD) affects over 33.9 million individuals worldwide and 5.69 million individuals in China (Jia et al., 2020). In addition to cognitive dysfunction, AD causes a variety of heterogeneous symptoms, which are defined as behavioral and psychological symptoms of dementia (BPSD), recently also known as neuropsychiatric symptoms (NPS) (Cerejeira et al., 2012). NPS include psychosis symptoms (e.g., delusions and hallucinations), affective symptoms (e.g., depression and anxiety), and hyperactivity (e.g., irritability, aggression, and euphoria) (van der Linde et al., 2014). Reports indicate that up to 80% of individuals with AD experienced greater severity and frequency of NPS compared to other forms of dementia (Lyketsos et al., 2002). A recent study reported that NPS also presented in preclinical stages of AD, including in individuals with subjective cognitive decline (SCD) with normal cognition and mild cognitive impairment (MCI) (Tsunoda et al., 2020). Notably, NPS may be a risk factor for the progression of the dementia stage of AD, including the preclinical stage (Peters et al., 2013; Burhanullah et al., 2020). Extensive evidence has indicated the association between NPS and poor outcomes, including high stress perception and decline of quality of life among patients and caregivers, unplanned hospitalizations, and an increase in socioeconomic burden (Feast et al., 2016; Toot et al., 2017; Hessler et al., 2018; Kim et al., 2021). Thus, alleviating NPS constitutes a critical component of AD management to improve the wellbeing of individuals with AD and their caregivers.

Currently, NPS management comprises pharmaceutical and non-pharmaceutical interventions. Drug therapy contributes to reducing short-term cognitive decline, but there is insufficient evidence on the efficacy of drug therapy for NPS (Fink et al., 2020). Non-pharmaceutical interventions predominantly involve psychosocial measures associated with risk factor management, such as music therapy and exercise (Raglio et al., 2015; Shi et al., 2020). Despite the effectiveness of several psychosocial interventions for NPS reported in the literature (Abraha et al., 2017; Hsu et al., 2017), no significant improvements have been observed in long-term care, and the dosage of multi-component interventions is challenging to measure and track (Orgeta et al., 2017; Yang et al., 2021). Several studies have investigated potential biological variables associated with NPS in AD to identify novel therapeutic interventions. For instance, 5-Hydroxytryptamine transporter gene-linked polymorphic region (5-HTTLPR) (Borroni et al., 2006; D'Onofrio et al., 2019), apolipoprotein E (D'Onofrio et al., 2011; Chen et al., 2012), and dopamine receptor polymorphisms (Proitsi et al., 2012) have been associated with one or more symptom groups of NPS. However, these biological variables are not feasible targets for modulation as therapeutic interventions for NPS.

The genetic and environmental risk factors associated with the onset and progression of AD are heterogeneous and sporadic. Emerging evidence suggests a link between AD and oral health, based on self-reported oral health-related quality of life (OHRQoL), objective indicators of oral health clinical assessments (e.g., missing teeth and gingival bleeding) (Ericsson et al., 2009; Zuluaga et al., 2012), and other indicators, including oral microbiota (Sureda et al., 2020), active potent neuroinflammatory regulators (e.g., IL-1β, IL-6, and TNF-α) (El Idrissi et al., 2021), and high salivary cortisol levels (Venturelli et al., 2016). An age-related change of oral status together with a decline in cognitive may be defined as a state of oral frailty (Dibello et al., 2021), representing toothache, oral dryness, tooth loss, dental caries, periodontal disease, and a set of worse oral daily practice functions, which may result in psychological distress (Vasiliou et al., 2016; Turner et al., 2017)and behavioral disturbances (Park et al., 2014; Kubo et al., 2017; Dahl et al., 2018). These oral clinical symptoms may eventually evolve into new stressors, thereby affecting disease outcomes (Mariño et al., 2020). To this end, several studies have explored the association between oral health and the onset and progression of AD. Nevertheless, there is a paucity of studies investigating the relationship between oral health and NPS. To our knowledge, there has only been one Japanese study (Fujihara et al., 2013) and a survey in the United Kingdom (Tsaroucha et al., 2015) that focused specifically on the association of NPS with dentures and oral-related symptoms. Nevertheless, these studies described the relationship between oral status and NPS based on a single aspect without combining related psychosocial, clinical, and biological indicators. Due to these deficiencies, it remains unclear whether and how specific oral-related indicators affect NPS in individuals with AD.

**Figure 1:**
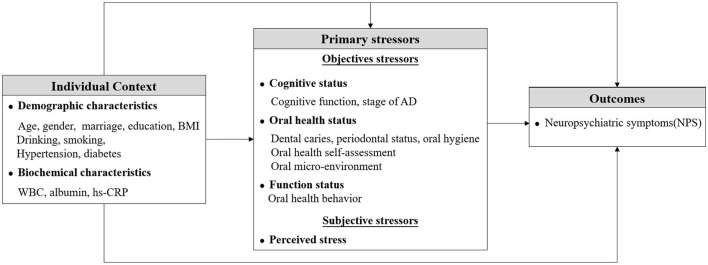
The conceptual framework of this study.

The aforementioned indicators of oral status, including persistent and gradually aggravated stimulation, are significantly related to dementia (Katz et al., 2010). Judge et al. (2010) proposed the stress process model (SPM), which conceptualizes and examines the illness experience of dementia. The model outlines key stressors in the illness experience from symptom onset to later stages of cognitive loss and posits relationships among stressors and outcomes (e.g., overall emotional, psychological, and physical reactions). Based on the SPM, this study regarded oral health-related indicators as primary stressors and divided them into subjective stressors [i.e., perceived stress (PS)] and objective stressors. Oral health-related indicators were considered as objective stressors, including clinical oral health assessments (i.e., dental caries, periodontal status, and oral hygiene), oral health self-assessments (self-reported physical and psychosocial function, pain, or discomfort), oral micro-environment, and oral health behavior. Based on this framework, this study aimed to comprehensively assess and clarify the relational networks between these primary stressors and the NPS of individuals with AD. We hypothesized that (a) there were disparities in oral status and NPS among elderly patients with AD progression, and (b) several oral health-related stressors may have direct and/or indirect effects on NPS in patients with AD, thereby serving as adjustable targets to guide interventions for NPS (Figure 1).

## Materials and Methods

### Study Design and Sampling

This cross-sectional survey was conducted in four communities in Chongqing Municipality from March 2021 to July 2021. The study protocol was approved by the Ethics Committee of the First Affiliated Hospital of Chongqing Medical University (Approval No. 2018–084) and was performed in accordance with the Declaration of Helsinki. Written informed consent was obtained from all participants or their legally authorized caregivers. A total of 106 participants were included, comprising patients diagnosed with SCD (*n* = 35), MCI (*n* = 36), or AD (*n* = 35). Participants were recruited through local community health service centers that offer a series of activities for older adults, including for example health education lectures, regular physical examination activities, or family visits, as well as through posters in local centers or local hospital memory clinics. Interested persons could contact us by emails, Wechat, or telephone shown in the posters. We also contacted probable targeted persons through family doctor team. The diagnosis of AD and MCI was based on the criteria of the National Institute of Neurological and Communication Disorders and Stroke and Alzheimer's Disease and Related Disorders Association (NINCDS-ADRDA) (Albert et al., 2011; McKhann et al., 2011). The diagnostic criteria for SCD referred to those proposed by Jessen in 2014 (Jessen, 2014) and China Pre-clinical Alliance for AD (Han, 2018). All diagnoses were performed by two experienced neurologists. Participants were enrolled in the study according to the inclusion and exclusion criteria. Participants in each group were aged from 65 to 90 years with a body mass index (BMI) in the normal range for the Chinese population (18.5–24.0 kg/m^2^) and were matched for sex and education (China Working Group on Obesity, 2004). Exclusion criteria were as follows: (a) unable to respond to the questionnaires or cooperate during oral assessment, (b) presented with oral diseases such as oral cancer and oral trauma, (c) had indwelling nasogastric tubes, (d) presented with severe heart and kidney diseases, (e) history of taking antibiotics within 1 month prior to the study, and (f) any kind of tumor or rheumatoid immune disease.

### Questionnaire Survey

Neuropsychological assessments, including the mini-mental state examination (MMSE), neuropsychiatric inventory questionnaire (NPI), and Clinical Dementia Rating (CDR) were performed by two experienced neurologists to determine diagnoses. NPI, consisting of sub-items of delusions, hallucinations, agitation, depression, anxiety, euphoria, apathy, disinhibition, irritability, abnormal motor behaviors, night-time behavior disturbances, appetite, and eating disturbances were administered to evaluate NPS (Leung et al., 2001). The demographic and clinical information included age, sex, marital status, education, smoking, drinking, hypertension, and diabetes. The weight and height of individuals were measured to calculate BMI. Additionally, the self-reported oral health status, oral health behavior, and PS were assessed using the geriatric oral health assessment index (GOHAI), oral health behavior questionnaire, and PS scale-10, respectively. The self-reported oral health status, conceptualized as OHRQoL, provides insight into the individuals' perception of their oral conditions, not only focusing on the damage of physiological function, but also the changes of psychological and social functions (Lee et al., 2007). The GOHAI and the Oral Health Impact Profile (OHIP)-14 are currently the most extensive OHRQoL assessment instruments (John et al., 2016). The GOHAI is a verified and straightforward instrument specially designed for older adults and widely used, providing reliable evidence of self-perceived OHRQoL concerning physical function, psychosocial function, and pain or discomfort. It is more sensitive to the detection of functional changes and is more suitable than OHIP-14 when evaluating the subjective perception of oral health and clinical changes (Locker et al., 2001). Details of the questionnaires are provided in Supplementary Table 1. All questionnaires were conducted by trained research staff *via* face-to-face interviews. Participants' relatives and/or caregivers were additionally interviewed to obtain more information.

### Dental Clinical Examination

Dental clinical examination, including dental caries, periodontal status, and oral hygiene were conducted by a professional stomatologist equipped with professional record personnel. All procedures and criteria were based on the fifth edition of the World Health Organization “Oral Health Surveys Basic Methods” (World Health Organization, 2021).

The dental caries experience of the participants was assessed using the DMFT index, comprising the sum of decayed (D), missing (M), and filled teeth (FT). The basis for DMFT calculations is 32 teeth, including wisdom teeth. The DT component includes all teeth with carious crowns, carious root, and/or filled crowns with caries. The MT component includes all missing teeth due to caries or for any other reason. The FT component includes teeth with filled crowns but without caries. Gingival bleeding (GB), periodontal pockets (PP), and loss of attachment (LoA) were measured using the WHO community periodontal index (CPI) periodontal probe and were considered indicators of periodontal status. These three indicators were recorded by dividing the mouth into sextants (sextant 1: 16, 17; sextant 2: 11; sextant 3: 26, 27; sextant 4: 36, 37; sextant 5: 31; and sextant 6: 46, 47). GB scores were recorded as 0 or 1 (absence or presence of bleeding, respectively). PP scores were recorded as 0 (absence of condition), 1 (pocket: 4–5 mm), or 2 (pocket: ≥6 mm). LoA scores were recorded as 0 (0–3 mm), 1 (4–5 mm), 2 (6–8 mm), 3 (9–11 mm), or 4 (≥12 mm). Oral hygiene was assessed using the Turesky modification of the Quigley-Hein Index to detect teeth around the cheek and tongue and to calculate the average dental plaque index (PLI) of the buccal and lingual sides of the upper and lower jaws (Turesky et al., 1970). PLI was scored from 0 to 5, with higher scores indicating poorer oral hygiene.

### Sample Collection and Analysis

Saliva samples were collected before oral examinations to avoid potential bleeding caused by improper oral examinations, which may have affected the results of saliva sample analysis. Participants were instructed to avoid eating or drinking anything other than water for 1 h before saliva collection between 9 and 11 o'clock in the mid-morning. Participants leaned forward above a sterile bottle placed on ice to allow saliva to flow naturally for collection of 1–2 mL of unstimulated saliva. The saliva was transferred to a frozen tube, placed in an icebox, and stored at −80°C for cryopreservation within 4 h.

### Enzyme-Linked Immunosorbent Assay (ELISA) Analysis

Enzyme-linked immunosorbent assay (ELISA) kits (Gianglai, China) were used to assay salivary pro-inflammatory cytokines (IL-1β, TNF-α, and IL-6), human cathepsin B (CTSB), and cortisol. All procedures were performed based on the manufacturer's instructions. The concentrations of these indicators were expressed by comparing the average absorbance readings of each sample with the concentration in the standard curve.

### 16srDNA Gene Sequencing

DNA from different samples was extracted using the E.Z.N.A. ®Stool DNA Kit (D4015, Omega, Inc., USA) according to the manufacturer's instructions. DNA extraction quality was determined using 2% agarose gel electrophoresis, and DNA was detected with an ultraviolet spectrophotometer. The 16srRNA gene (V3-V4) region (Logue et al., 2016) was amplified using primers 341F (5′-CCTACGGGNGGCWGCAG-3′) and 805R (5′-GACTACHVGGGTATCTAATCC-3′) using a PCR assay. The PCR products were purified with AMPure XT beads (Beckman Coulter Genomics, Danvers, MA, USA) and quantified using Qubit (Invitrogen, USA). The amplicon pools were prepared for sequencing, and the size and number of the amplicon library were evaluated using Agilent 2100Bioanalyzer (Agilent, USA) and Illumina Library Quantification Kit (Kapa Biosciences, Woburn, MA, USA), respectively. The libraries were sequenced on the NovaSep PE250 platform according to the manufacturer's recommendations.

### Venous Blood Sample Collection

Venous blood samples were collected into vacutainer tubes containing EDTA anticoagulants by professional research cooperators in local community health service centers. Within 24 h, white blood cells (WBCs), hypersensitive C-reactive protein (hs-CRP), and albumin (ALB) were tested by the same doctor in the laboratory department of each community health service center. All procedures and operations were completed in accordance with the local center's routine management protocols.

### Statistical Analysis

Indices of bioinformatics analysis were calculated using QIIME2, and the graphs were drawn with the R package (Bolyen et al., 2019). Paired-end reads were merged using the Fast Length Adjustment of SHort reads (FLASH) tool. Quality filtering on the raw reads was performed under specific filtering conditions to obtain high-quality clean tags according to fqtrim (v0.94). Chimeric sequences were filtered using Vsearch software (v2.3.4). All reads were deposited and grouped into features at a sequence identity of 100% similarity clustering. After dereplication using DADA2 (Callahan et al., 2016), we obtained the feature table and feature sequence. Alpha diversity (a measure of sample complexity) was estimated using four indices (Chao1, Observed species, Shannon, and Simpson). The Kruskal–Wallis test and Mann–Whitney U test were performed to assess the differences in alpha diversity among three groups and between two groups, respectively. Bacterial diversity between samples was evaluated using beta diversity by principal coordinate analysis (PCoA) with weighted uniFrac and unweighted uniFrac distance matrix. Significant between-group differences in sample species based on relative abundance were analyzed using the Kruskal–Wallis test for multi-group comparisons and Mann–Whitney U test for two-group comparisons.

Epidata 3.1 was used for data input and SPSS version 23.0 was used for data analysis. Descriptive analyses of variables were presented as percentages for categorical variables, means (*M*) and standard deviations (*SD*) for continuous variables with normal distribution, and interquartile ranges (IQRs) for skewed data. The normality of data distribution was assessed using the PP-plot. Intergroup comparisons were performed using the chi-squared test or Fisher's exact test for categorical variables and the Kruskal–Wallis test or one-way ANOVA for continuous variables. Bonferroni *post-hoc* test was applied to test pairwise multiple comparisons. Predictors of PS and NPS were determined using multiple stepwise linear regression analyses. Variables with *P* < 0.05 in univariate regression analysis were included in the model. The fit of the multivariate linear regression analysis model was tested using model-residual and normal-probability graphs.

A structural equation model (SEM) was established using AMOS 17.0 to test the mediating role of PS between oral health-related stressors and NPS. The mediation effect was analyzed with bias-corrected Bootstrap using 2000 iterations, which is a recommended test of indirect effects (Hayes, 2009). The 95% confidence interval (CI) of the estimated value did not contain 0, indicating that part of the mediation effect was valid. Model fitting with a Chi-square value/degrees of freedom ratio (χ^2^/df) <3.0, RMSEA < 0.07 (Miller et al., 2020), GFI, IFI, AGFI, and CFI ≥ 0.90 are typically regarded as an indication of good coordination (Maccallum et al., 1996). Statistical significance was set at a two-tailed α-value of 0.05.

The sample size was computed by conducting linear multiple regression *post-hoc* to power analysis using the G^*^ Power 3.1 software (Beck, 2013). Power was over 0.98 based on a sample size of 106 an alpha error of 0.05, a medium effect size of 0.15 and a number of contained predictors of 7. Based on sample size estimation of SEM analysis (Kendall et al., 1994), samples need to be larger than 10 times the number of estimated parameters. In this study, SEM included 5 parameters and sample of 106 satisfied the requirements.

## Results

### Demographic and Clinical Characteristics of Participants With SCD, MCI, and AD

**Figure 2:**
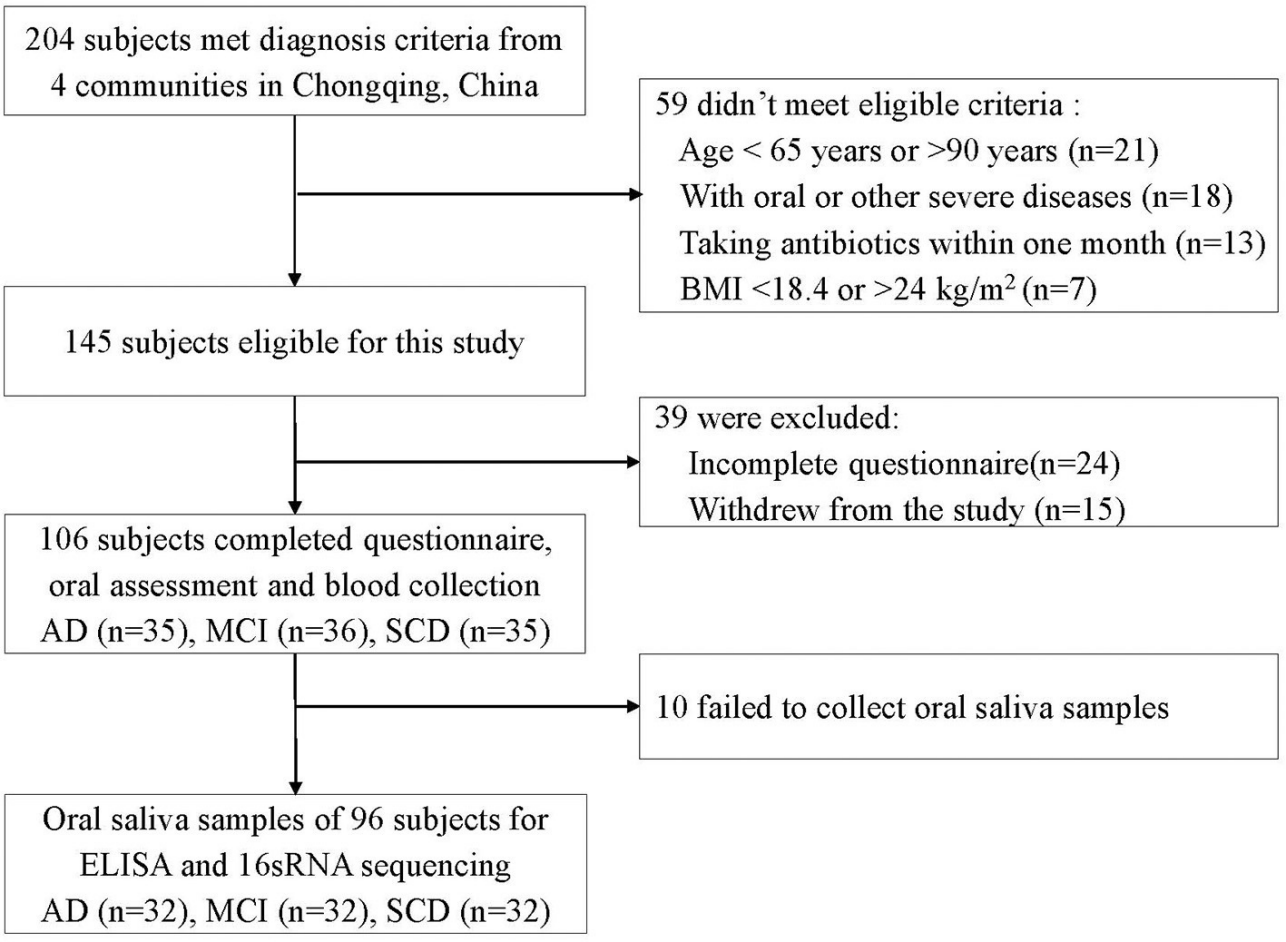
Flow chart of participants.

In total, 204 participants met the diagnostic criteria and 145 participants were eligible for enrolment in this study after screening for inclusion criteria. One hundred and six participants completed all questionnaires, dental clinical examination, and blood collection. Oral saliva samples were collected from 96 participants (Figure 2). As shown in Table 1, no significant differences were observed in age, sex, marital status, education, BMI, smoking, drinking, hs-CRP levels, WBC counts, and serum albumin among SCD, MCI, and AD groups (*P* > 0.05). MMSE scores were significantly lower in the AD group than in the SCD and MCI groups (*P* < 0.001 for both comparisons), and significantly lower in the SCD group than in the MCI group (*P* < 0.001). NPS was severer in the AD and MCI groups than in the SCD group (*P* < 0.001, *P* = 0.002, respectively).

**Table 1:** Demographic, clinical and biochemical characteristics of individuals.

**Characteristics**	**Total**	**SCD (*n* = 35)**	**MCI (*n* = 36)**	**AD (*n* =35)**	***P***	***Post-hoc***
Age (mean ± SD)	75.30 ± 6.63	74.11 ± 6.25	75.42 ± 7.77	76.37 ± 5.67	0.364^a^	n.s.
Female, n (%)	40 (37.7)	13 (37.1)	14 (38.9)	13 (37.1)	0.985^b^	n.s.
Married, n (%)	28 (26.4)	12 (34.3)	10 (27.8)	6 (17.1)	0.260^b^	n.s.
Education less than middle school, n (%)	59 (55.7)	16 (45.7)	21 (58.3)	22 (62.9)	0.326^b^	n.s.
BMI (mean ± SD)	21.96 ± 2.18	22.10 ± 2.33	21.84 ± 2.16	21.93 ± 2.11	0.879^a^	n.s.
Smoking, n (%)	23 (21/7)	8 (22.9)	8 (22.2)	7 (20.0)	0.955^b^	n.s.
Drinking, n (%)	26 (24.5)	8 (22.9)	9 (25.0)	9 (25.7)	0.959^b^	n.s.
Hypertension, n (%)	56 (52.8)	23 (65.7)	17 (47.2)	16 (45.7)	0.174^b^	n.s.
Diabetes, *n* (%)	35 (33.0)	14 (40.0)	9 (25.0)	12 (34.3)	0.398^b^	n.s.
hs-CRP ≥ 1.0 mg/L, *n* (%)	34 (32.1)	8 (22.9)	14 (38.9)	12 (34.3)	0.331^b^	n.s.
WBC, 10^9^/L, (mean ± SD)	5.70 ± 1.58	5.53 ± 1.51	5.59 ± 1.35	5.98 ± 1.84	0.431^a^	n.s.
Albumin, g/L, (mean ± SD)	40.69 ± 3.60	41.89 ± 4.22	40.06 ± 3.06	40.14 ± 3.23	0.053^a^	n.s.
MMSE (mean ± SD)	22.42 ± 5.49	27.69 ± 1.21	23.17 ± 2.67	16.40 ± 4.24	<0.001^a^	SCD < MCI < AD
NPS, median (IQR)	6 (0, 21.3)	0 (0, 4.0)	14 (0, 22.0)	20 (10, 26.0)	<0.001^c^	SCD < MCI, SCD < AD

a *One-way ANOVA*.

b *Chi-squared test*.

c *Kruskal-Wallis test*.

*SD, standard deviation; BMI, body mass index; hs-CRP, high sensitivity C-reactive protein; WBC, white blood cell, MMSE, mini-mental state examination; NPS, neuropsychiatric symptoms; IQR, interquartile range; n.s., not significant*.

### Comparison of Primary Stressors Among SCD, MCI, and AD Groups

Table 2 presents a comparison of objective and subjective stressors among SCD, MCI, and AD groups. Significant differences were observed in indicators of oral health status, including DMFT, MT, FT, PLI, and LoA, among the three groups (*P* < 0.05). *Post-hoc* analysis revealed that DMFT scores (*P* < 0.001) and MT (*P* = 0.001) were significantly higher in the AD group than in the SCD group. FT scores (*P* = 0.032) were significantly higher in the MCI group than in the SCD group. PLI and LoA scores were significantly higher in the AD and MCI groups than in the SCD group (*P* < 0.001 for all comparisons). Further, LoA scores were significantly higher in the MCI group than in the SCD group (*P* = 0.006). In the self-assessment of oral health status, significant differences were observed in GOHAI total score and scores of sub-dimensions (i.e., physical function, pain or discomfort) among the three groups (*P* < 0.001, *P* = 0.022, *P* < 0.001, respectively), whereby scores were significantly lower in the AD group than in the SCD group. Oral health behavior was better in the SCD group than in the AD group (*P* = 0.010).

**Table 2:** Comparation of primary stressors among individuals with SCD, MCI, and AD.

**Variables**	**Total**	**SCD (*n* = 35)**	**MCI (*n* = 36)**	**AD (*n* = 35)**	***P***	***Post-hoc***
DMFT	23.5 (14.0, 28.0)	17.0 (13.0,24.0)	24.0 (14.0, 29.0)	27.0 (23.0, 32.0)	<0.001^a^	SCD < AD
DT	7.5 (4.0, 12.3)	7.0 (4.0, 10.0)	8.5 (4.5, 13.7)	8.0 (2.0, 15.0)	0.725^a^	n.s.
MT	10.0 (5.0, 19.0)	8.0 (4.0, 14.0)	10.5 (4.3, 18.5)	17.0 (10.0, 25.0)	0.001^a^	SCD < AD
FT, *n* (%)					0.032^b^	SCD < MCI
0	87 (82.1)	24 (68.6)	33 (91.67)	30 (85.7)		
≥1	19 (17.9)	11 (31.4)	3 (8.33)	5 (14.3)		
PLI^#^	3.57 ± 1.02	2.98 ± 0.84	3.72 ± 1.10	4.08 ± 0.78^#^	<0.001^c^	SCD < AD, SCD < MCI
LoA^#^	2.84 ± 1.06	2.20 ± 0.83	2.89 ± 0.98	3.53 ± 0.94	<0.001^c^	SCD < MCI < AD
GB, *n* (%)^#^	94 (93.1)	33 (94.3)	34 (94.4)	27 (90.0)	0.789^d^	
PP, *n* (%) ^#^					0.490 ^b^	n.s.
0–3 mm	15 (14.9)	6 (17.1)	4 (11.1)	5 (16.7)		
4–5 mm	58(57.4)	23 (65.7)	20 (55.6)	15 (50.0)		
≥6 mm	28 (27.7)	6 (17.1)	12 (33.3)	10 (33.3)		
GOHAI	40.04 ± 4.36	42.31 ± 4.10	39.69 ± 3.90	38.11 ± 4.12	<0.001^c^	SCD > AD, SCD > MCI
Physical function	11.73 ± 2.89	12.63 ± 3.15	11.81 ± 2.80	10.74 ± 2.43	0.022^c^	SCD > AD
Psychosocial function	18.72 ± 1.69	18.83 ± 1.92	18.94 ± 1.58	18.37 ± 1.54	0.324^c^	n.s.
Pain or discomfort	9.47 ± 1.72	10.86 ± 1.22	8.83 ± 1.63	8.74 ± 1.42	<0.001^c^	SCD > AD, SCD > MCI
Oral health behavior	21.26 ± 6.81	23.74 ± 6.74	21.17 ± 6.31	18.89 ± 6.67	0.010	SCD > AD
CTSB, ng/ml, median (IQR)^¶^	28.6 (23.7, 36.2)	24.2 (20.2, 27.2)	31.4 (26.6, 37.0)	34.7 (28.9, 38.7)	<0.001^a^	SCD < AD, SCD < MCI
IL-1β, pg/ml, median (IQR)^¶^	52.7 (41.2, 62.7)	40.7 (33.5, 49.1)	56.4 (48.1, 63.0)	61.9 (55.5, 68.1)	<0.001^a^	SCD < AD, SCD < MCI
IL-6, pg/ml, median (IQR)^¶^	34.3 (27.7, 41.4)	31.5 (26.3, 39.6)	34.2 (27.3, 39.8)	40.9 (28.9, 45.5)	0.011^a^	SCD < AD
TNF-α, pg/ml, median (IQR)^¶^	56.7 (41.8, 64.0)	42.5 (38.1, 57.2)	57.0 (50.7, 65.6)	62.3 (53.8, 75.4)	<0.001^a^	SCD < AD, SCD < MCI
Cortisol, nmol/ml, median (IQR)^¶^	9.9 (8.4, 11.2)	8.2 (7.1, 9.8)	9.9 (8.6, 11.3)	11.1 (9.9, 12.0)	<0.001^a^	SCD < AD, SCD < MCI
PS (mean ± SD)	20.53 ± 3.03	18.74 ± 2.89	20.53 ± 2.87	22.31 ± 2.22	<0.001^c^	SCD < MCI < AD

a *Kruskal-Wallis test*.

b *Chi-squared test*.

c *One-way ANOVA*.

d *Fisher exact test*.

# *Five older adults in the AD group without teeth were missing*.

¶ *Sample of each group was 32 participants*.

*DMFT, decaying (D), missing (M), and filled (F) teeth index; PLI, plaque index; LoA, loss of attachment; GB, gingival bleeding; PP, periodontal pockets; GOHAI, the geriatric oral health assessment index; PS, perceived stress; CTSB, human cathepsin B; IL, Interleukin; TNF, tumor necrosis factor; n.s., not significant*.

**Figure 3:**
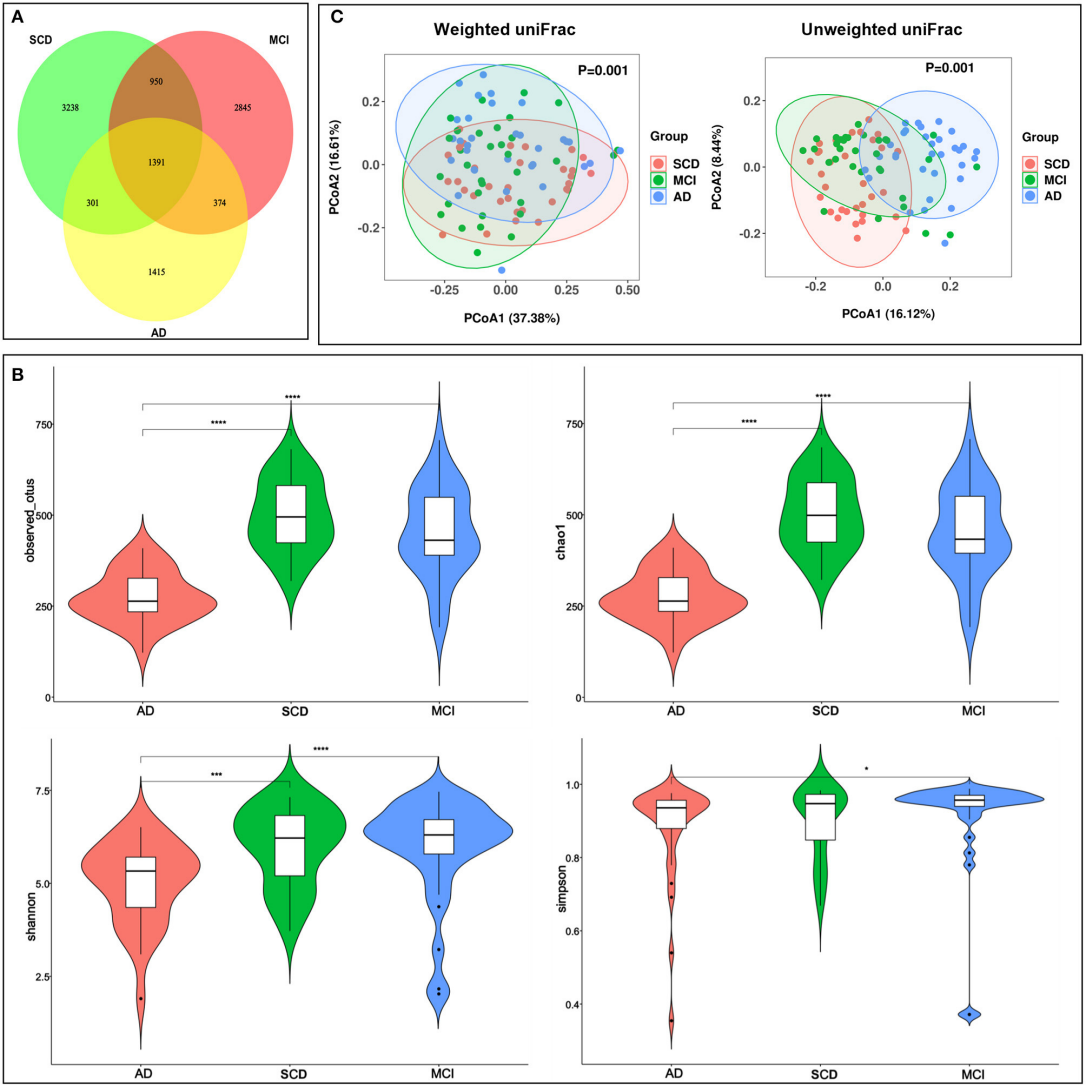
Bacterial diversity of the oral microbiota of SCD, MCI and AD groups. **(A)** A feature venn diagram among three groups. **(B)** The α-diversity of the oral microbiome among three groups according to observed-otus, Chao 1, Shannon index, and Simpson index. **(C)** The beta diversity was calculated using weighted (*P* = 0.002) and unweighted UniFrac (*P* = 0.001) by PCoA. ^****^P < 0.0001, ^***^P < 0.001, ^*^P < 0.05.

Comparison of the oral micro-environment among the three groups revealed that levels of the oral pro-inflammatory cytokines CTSB, IL-1β, and TNF-α were significantly higher in the AD and MCI groups than in the SCD group (*P* < 0.001 for all comparisons). IL-6 levels were significantly different between AD and SCD groups (*P* = 0.011). Oral microbiota were assessed using 16S rRNA MiSeq sequencing. A total of 6,890,504 high-quality 16S rRNA gene sequences were identified. As shown in the Venn diagram (Figure 3A), 1,391 features of the total 10,514 features were shared among all samples. Alpha diversity analysis (Figure 3B) revealed that compared to the MCI and SD groups, the AD group had significantly lower diversity as indicated by reduced Observed species, Chao1, and Shannon indices. These indices were not significantly different between MCI and SCD groups. Beta diversity analysis (Figure 3C) was used to display the microbiome space using PCoA. Both weighted and unweighted uniFrac results revealed significant differences among the three groups. The differences among the three groups of the top 10 species in relative abundance at phylum and genus levels were analyzed (Supplementary Table 2). *Firmicutes* and *Actinobacteria* phyla exhibited increased abundance in both AD group and MCI group than in the SCD group (*P* < 0.05 for all comparisons), whereas *Patescibacteria* and *Synergistetes* exhibited a gradually decreasing abundance among the three groups (AD < MCI < SCD, *P* < 0.001). These alterations were significantly different in both three-group and *post-hoc* comparisons. At the genus level, the abundance of *Porphyromonas* and *Prevotella* was significantly lower in the AD group than in the MCI and SCD groups (*P* < 0.05 for all comparisons).

With regard to subjective stressors, the highest and lowest PS scores were observed in the AD and SCD groups, respectively (SCD < MCI < AD, *P* < 0.001); these differences were statistically significant. In addition, the level of salivary cortisol, an objective physiological indicator of stress, displayed similar trends to those of PS.

### Factors Associated With NPS and PS

Linear regression analyses were conducted to examine the influencing factors associated with NPS and PS. All independent variables with *p* < 0.05 in simple linear regression were entered into the regression model by stepwise regression. As shown in Table 3, higher PS (β = 0.33, *P* < 0.001), PLI (β = 0.27, *P* < 0.001), LoA (β = 0.14 *P* = 0.033), and MT scores (β = 0.15, *P* = 0.005) were associated with worse NPS. Less pain or discomfort (higher scores) indicated a greater level of oral health as indicated in the self-assessment, which was associated with better NPS (β = −0.18, *P* = 0.003). Table 4 shows the linear regression model predicting PS in participants. PLI (β = 0.20, *P* = 0.044) and LoA (β = 0.28, *P* = 0.008) were positively associated with PS. Poorer performance in psychosocial function, a sub-dimension of GOHAI, also contributed to greater PS (β = −0.30, *P* < 0.001). However, in the multivariate analysis, oral pro-inflammatory cytokines (i.e., TNF-α) and salivary microbiota (i.e., *Actinobacteria*) were not significantly correlated with NPS and PS, although these factors were significant in the simple linear regression analysis.

**Table 3:** Analysis of the influencing factors of NPS among participants.

**Variables**	**Unstandardized coefficients**		**Standardized coefficients**	***t***	***P***	**95%** ***CI*** **of** ***B***	
	***B***	***S.E*.**	**β**			**Lower**	**Upper**
PS	1.15	0.21	0.33	5.365	<0.001	0.72	1.57
PLI	2.77	0.63	0.27	4.361	<0.001	1.50	4.03
LoA	1.47	0.68	0.14	2.172	0.033	0.12	2.82
Pain or discomfort of GOHAI	−1.15	0.38	−0.18	−3.056	0.003	−1.90	−0.40
MMSE	−0.32	0.18	−0.17	−1.802	0.075	−0.68	0.03
MT	0.19	0.07	0.15	2.873	0.005	0.06	0.32
**Disease stage (ref:SCD)**							
MCI	0.53	1.46	0.02	0.364	0.717	−2.37	3.43
AD	0.78	2.22	0.03	0.351	0.726	−3.64	5.20

*CI, confidence interval; PS, perceived stress; PLI, plaque index; LoA, loss of attachment; MMSE, mini-mental state examination; MT, missing teeth; GOHAI, the geriatric oral health assessment index*.

**Table 4:** Analysis of the influencing factors of PS among participants.

**Variables**	**Unstandardized coefficients**		**Standardized coefficients**	***t***	***P***	**95%** ***CI*** **of** ***B***	
	***B***	***S.E*.**	**β**			**Lower**	**Upper**
PLI	0.60	0.29	0.20	2.043	0.044	0.02	1.18
LoA	0.83	0.30	0.28	2.733	0.008	0.23	1.44
Psychosocial function of GOHAI	−0.54	0.14	−0.30	−3.734	<0.001	−0.82	−0.25
MMSE	−0.04	0.08	−0.08	−0.525	0.601	−0.21	0.12
**Disease stage(ref:SCD)**							
MCI	0.79	0.65	0.13	1.223	0.225	−0.50	2.08
AD	1.35	1.04	0.20	1.296	0.199	−0.72	3.42

*CI, confidence interval; PS, perceived stress; PLI, plaque index; LoA, loss of attachment; GOHAI, the geriatric oral health assessment index; MMSE, mini-mental state examination*.

### Effect Analysis of the Mediation Model

**Figure 4:**
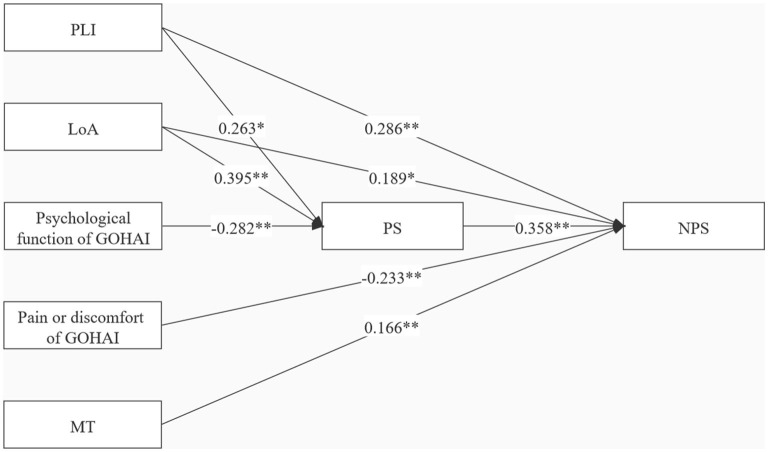
Specific indirect effects in the mediation model. Path coefficients were standardized estimates. PLI, plaque index; LoA, loss of attachment; MT, missing teeth; GOHAI, the geriatric oral health assessment index; PS, perceived stress; NPS, neuropsychiatric symptoms; ^**^P < 0.001; ^*^P < 0.05.

An in-depth analysis was performed to explore the mediating effect of PS between oral health-related stressors and NPS. The hypothetical path analysis model was established based on the theoretical framework model (Figure 1) and the results of the multi-regression analysis (Tables 3, 4). PLI, LoA, psychological function, pain or discomfort, and MT were defined as exogenous variables, whereas PS and NPS were defined as endogenous variables. All paths were significant in the model, as shown in Figure 4. Overall, the path model-fit indices were as follows: χ^2^/*df* = 1.232, GFI = 0.990, CFI = 0.998, NFI = 0.989, TLI = 0.985, and *RMSEA* = 0.048, demonstrating an excellent model fit. Table 5 presents the direct, indirect, and total effects of specific paths. NPS was directly affected by PLI (β = 0.286, *Bootstrap 95% CI* = 1.779~4.203), LoA (β = 0.189, *Bootstrap 95% CI* = 0.356~3.194), pain or discomfort (β = −0.233, *Bootstrap 95% CI* = −2.042~−0.834), MT (β = 0.166, *Bootstrap 95% CI* = 0.095~0.368), and PS (β = 0.358, *Bootstrap 95% CI* = 0.846~1.637). Notably, indirect effects of PLI (β = 0.094, *Bootstrap 95% CI* = 0.381~1.813) and LoA (β = 0.141, *Bootstrap 95% CI* = 0.710~2.537) on NPS mediated by PS were verified in this model, with mediation proportion accounting for 24.74% and 42.73%, respectively. Psychological function (β = −0.101, Bootstrap 95% CI = −1.002~−0.316) exerted indirect effects on NPS *via* PS, indicative of a complete mediation effect. Additionally, PLI (β = 0.263, *Bootstrap 95% CI* = 0.295~1.813), LoA (β = 0.395, *Bootstrap 95% CI* = 0.632~1.813), and psychological function (β = −0.282, *Bootstrap 95% CI* = −0.752~−0.275) were direct factors affecting PS.

**Table 5:** Standardized direct, indirect and total effects of the variables.

**Pathways**	**Effect relationship**	**β**	**Effect proportion(%)**	**Bootstrap 95%CI**	
				**Lower**	**Upper**
PLI → NPS	Direct effects	0.286	75.26	1.779	4.203
	Indirect effects	0.094	24.74	0.381	1.813
	Total effects	0.380	100.00	2.723	5.236
LoA → NPS	Direct effects	0.189	57.27	0.356	3.194
	Indirect effects	0.141	42.73	0.710	2.537
	Total effects	0.330	100	1.869	4.537
Psychological function → NPS	Indirect effects	−0.101	100	−1.002	−0.316
	Total effects	−0.101	100	−1.002	−0.316
Pain or discomfort → NPS	Direct effects	−0.233	100	−2.042	−0.834
MT → NPS	Direct effects	0.166	100	0.095	0.368
PS → NPS	Direct effects	0.358	100	0.846	1.637
PLI → PS	Direct effects	0.263	100	0.295	1.813
LoA → PS	Direct effects	0.395	100	0.632	1.813
Psychological function → PS	Direct effects	−0.282	100	−0.752	−0.275

*PLI, plaque index; NPS, neuropsychiatric symptoms; LoA, loss of attachment; MT, missing teeth; PS, perceived stress*.

## Discussion

To our knowledge, this is the first study based on SPM to comprehensively assess oral health status and evaluate possible associations and influencing pathways between oral health-related stressors, PS, and NPS among Chinese community-dwelling individuals with AD, MCI, and SCD. The present study demonstrated that individuals with AD experienced poorer oral health, greater stress perception, and worse NPS compared to individuals in the preclinical stage of AD. Additionally, objective stressors, including dental caries (i.e., MT), periodontal status (i.e., LoA), oral hygiene (i.e., PLI), pain or discomfort on GOHAI, and subjective stressors (i.e., PS) contributed to NPS, in which LoA and PLI were identified as predictors of NPS mediated by PS. These findings validate our study hypotheses and afford novel perspectives on oral health indicators as adjustable targets to guide therapeutic interventions for NPS in individuals with dementia based on the SPM.

The oral micro-environment, clinical oral health assessment, oral health self-assessment, and oral health behavior assessed in this study are putatively related to AD. With regard to the oral micro-environment of participants in our study, levels of pro-inflammatory cytokines (e.g., IL-1β, IL-6, and TNF-α) were significantly higher in the AD group than in the preclinical stage of AD groups, in accordance with previous studies (Bathini et al., 2020). Extant literature (Liu et al., 2019; Holmer et al., 2021) suggests that the diversity and richness of microbiota are lower in individuals with AD, and microbial biomarkers (e.g., *Lautropia, Moraxella*, etc.) are able to distinguish AD from other stages of AD and/or healthy individuals. Our results are in agreement with these findings. Some studies have revealed that oral microbiota was found in AD brains of autopsy cases and was presented higher density and far greater variety than in the cognitively normal groups, which was evidenced the entry of oral microbiota into the brain and the decline progression of cognitive function (Miklossy, 2011; Dominy et al., 2019). Of note, *Firmicutes*, the bacteria with highest relative abundance, was particularly abundant in AD and MCI groups compared to the SCD group in this study. *Firmicutes* has been reported to be the predominant phylum of dental plaques and is associated with tooth loss (Wu et al., 2021). The genera of *Firmicutes* (i.e., *Streptococcus*) may directly develop amyloid fibers and cause amyloid formation by the cell-surface localized adhesin antigen P1, leading to the prominent features of AD (Oli et al., 2012). Consistent with Wu's report, we also found AD group suffered poor oral hygiene, worse dental caries, and reduction of the number of teeth compared with the other groups (Wu et al., 2021). It was increasingly reported that dysbiosis of microbiota was associated with psychological outcomes, such as anxiety and/or depression behavior (Foster and McVey Neufeld, 2013; Johnson and Foster, 2018). Although there was no direct relationship between oral microbiota and mental disorder, instead, AD group showed worse oral health behavior and stress in our study. These studies mentioned above suggested that alterations of the oral microbiota might be the common risk factors of deterioration of oral health and progression of AD. The causal relationship between oral microbiota and psychological outcomes of AD needs more future studies.

An *in vitro* study reported that IL-1β and TNF-α increased the permeability of the blood-brain barrier (BBB) and promoted entry of periodontal pathogens into the brain, triggering neuroinflammatory responses and increased Aβ production (Dominy et al., 2019). “Dysbiosis” of the oral micro-environment in individuals with AD may promote cognitive decline and is supported by our findings. Numerous studies have indicated that alterations of the oral micro-environment predominantly cause caries, periodontal disease, and MT in the aged population (Dominy et al., 2019; Gao et al., 2020; Thomson and Barak, 2021). Further, we also observed that individuals with AD experienced worse dental caries, periodontal status, and oral hygiene, and poorer oral health behaviors with AD progression. As reported by Torales et al. (2017), individuals with mental disorders may experience the most frequent dental cavities and periodontal disease due to loss of interest in self-care, negative attitudes toward healthcare providers, and lack of cooperation in treatments. Krom et al. (2014). reported that changes in physiological, hormonal, and behavioral modifications could in turn modify the oral microbial population, contributing to tau hyperphosphorylation and accumulation of Aβ plaques, thereby promoting the onset or progression of AD (Sureda et al., 2020). Although the exact mechanisms underlying the link between oral bio-environment and AD remain unclear, it is hypothesized that “dysbiosis” of oral status in AD individuals forms a vicious cycle that promotes cognitive decline and oral disease.

We observed that NPS incidence and severity at different stages of AD were fairly consistent with the variation in trends of oral health status. Given the proposed relationship between oral health “dysbiosis,” worse NPS, and greater PS based on the conceptual framework originating from SPM, this study further explored the relationships of these potential stressors with NPS. Fujihara et al. (2013) reported lower oral activity of daily living scores and MT associated with aggressiveness and activity disturbances, and worse NPS in individuals with vascular dementia. The present study included more comprehensive variables compared to previous studies and demonstrated that indicators of oral health-related stressors, i.e., PLI, LoA, MT, and pain or discomfort of GOHAI, were significantly associated with worse NPS. These findings suggest that dental caries, periodontal disease, and oral hygiene may affect NPS, which provides novel insights into potential approaches for NPS management. A systematic review identified that increased dental decay based on DMFT scores and tooth loss were associated with psychiatric diagnoses (i.e., depression and anxiety) and proposed a closer collaboration with dental practitioners to promote physical health of psychiatric patients (Kisely et al., 2016). Contrary to our findings, a study in the United Kingdom did not identify a significant relationship between dental pain and NPS among elderly residents in nursing homes (Tsaroucha et al., 2015). A potential reason for these discrepancies could be due to differences in sample selection and oral pain assessment. Indeed, nearly two-thirds of British participants had no teeth (sans teeth indicate sans pain based on the assessment tool used and is indicative of minimal oral problems), while participants in our study had severe caries and periodontitis and only 5 out of 106 did not have teeth.

Compared with MCI and SCD groups, the AD group in this study reported greater PS, in accordance with salivary cortisol test results. Considerable evidence suggests that individuals experience more stress disorders with the diagnosis and progression of AD (Gradus et al., 2019; Sharp, 2019). In actuality, the sources of stress are diverse, such as oral health-related stressors mentioned in this study and other factors including cognitive abilities (Guerdoux-Ninot and Trouillet, 2019), poor social support (Yang et al., 2021), and comorbidities (Moazzami et al., 2021). Thus, we used PSS-10 to assess PS, a scale that does not link an assessment to a specific situation; rather, this scale encompasses all situations in daily life that are considered stressful, as well as their reactions. As a subjective stressor, PS exhibited a strong positive relationship with NPS. A scoping review identified determinants of specific NPS (e.g., aggression, agitation, apathy, depression, and psychosis) from patient, caregiver, and environmental aspects. However, few of the included studies focused on the effects of PS on NPS (Kolanowski et al., 2017). Our findings provide novel insight into potential interventions based on targeted risk factors to manage NPS.

Previous studies have reported a significant association between PS and oral disease in older adults (Hilgert et al., 2006; Ishisaka et al., 2007). We further explored the potential link between oral health-related stressors and PS. LoA, PLI, and psychosocial discomfort in the GOHAI were significantly associated with PS. In contrast, MMSE and clinic disease stages were not significantly associated with PS, suggesting that poorer oral status and psychosocial function may play more important roles in PS among these individuals. Further, mediation model analysis confirmed the mediating role of PS between oral health and NPS. PLI and LoA affected NPS directly and also indirectly affected NPS *via* PS, while psychological discomfort in the GOHAI indirectly affected NPS *via* PS. These results indicate that elderly individuals with severe dental cavities and chronic periodontal disease may experience self-reported psychological discomfort that affects PS. These factors may reciprocally interact and jointly contribute to NPS. Accordingly, preventive interventions such as regular dental visits, effective tooth brushing, and timely treatment of dental problems may be necessary for individuals with AD to reduce their risk of dental caries and periodontal and oral mucosal diseases. We also found some challenges in the process of dental clinical examination in non-gerodontologic settings, in accordance with a study conducted in primary care (Mettes et al., 2008). Dental clinical assessment should be performed gently and promptly by skilled dental general practitioners due to the special conditions of AD individuals, for instance, ineffective doctor-patient communication, poor cooperation, and tolerance for inspection. Moreover, settings of the dental examination were required to be a quiet, independent space with comfortable equipment, which would reduce the irritation and improve the satisfaction of AD individuals. In the present study, dental clinical assessment was conducted in the dental clinic of the local community health service centers. Nevertheless, specialized dental examination services are relatively unavailable in community health centers, especially in rural areas of western China. Additionally, ignorance of oral health and insufficient cognition among older adults are also obstacles hindering dental examination, thereby promoting awareness and intention of dental attendance are necessary (Åstrøm et al., 2018).

### Strengths and Limitations

Although the associations between oral health and cognitive status have been explored, there is a paucity of studies examining the effects of oral health on NPS, especially based on a complete theoretical model of psychology. This study, based on the SPM for individuals with AD, is the first to comprehensively examine the relationship of objective oral health-related stressors and subjective stressors (i.e., PS) with NPS. Further, we performed an in-depth exploration of relevant pathways and the extent to which oral health-related stressors affected NPS mediated by PS using mediation model analysis.

Several limitations should be addressed when interpreting the findings of this study. The diagnosis of AD was established as per the revised NINCDS-ADRDA criteria (Dubois et al., 2014), without performing cerebrospinal fluid assays and/or positron emission tomography (PET) imaging due to the prohibitive nature of invasive or expensive examination methods in older adults. Nevertheless, the revised NINCDS-ADRDA criteria have been used extensively for population-based investigations, and its reliability has been verified (de Jager et al., 2010). Furthermore, although we first observed the effect of oral health on NPS and the mediating effect of PS based on cross-sectional data, future studies should examine whether they promote the occurrence and severity of NPS and the underlying mechanisms. Additionally, the association between oral health-related stressors and specific symptom clusters of NPS, such as aggression and psychosis symptoms, was not further analyzed due to sample size limitations. Because of the various biological, neuropsychological, and oral clinical assessments involved, collecting large sample data with limited time and resources is a challenge. Nevertheless, we improved sample representativeness by matching general and clinical data from different groups of samples. Moreover, the results of inter-group comparisons of these variables did not exhibit any significant differences. Ultimately, interpretation of the salivary cytokine data should be considered carefully since the salivary flow rate was not calculated in this study. Age was considered an important factor to affect saliva production, fortunately, participants were aged-matched with no significant difference and each saliva sample was collected within 6 min in this study (Szabo and Slavish, 2021). In addition, the literature revealed that some cytokines (e.g., IL-1β, CRP) appear influenced by flow rate, whereas others do not (e.g., IL-10, IL-6) (Szabo and Slavish, 2021). Future studies may need to determine which specific salivary cytokines are affected by salivary flow rate.

## Conclusion

Given the growth in the aging population worldwide, an increasing incidence of AD and oral health problems may be expected. This may impact disease-related distress and quality of life. Although there are currently no effective treatments for AD, the relationship between oral-derived stressors, PS, and NPS is modifiable, as indicated by the findings of this study. A deeper understanding of the predictive role of oral health indicators, such as PLI and LoA, in the relationship between PS and NPS may facilitate the development of more effective interventions that can address oral disease and provide adequate social support in individuals with NPS.

## Data Availability Statement

The datasets presented in this study can be found in online repositories. The names of the repository/repositories and accession number(s) can be found below: https://www.ncbi.nlm.nih.gov/bioproject/PRJNA774447.

## Ethics Statement

The studies involving human participants were reviewed and approved by the Ethics Committee of the First Affiliated Hospital of Chongqing Medical University (Approval No. 2018–084). The patients/participants provided their written informed consent to participate in this study.

## Author Contributions

BY, QZ, and JW performed substantial contributions to conception and design, interpretation of data, and manuscript drafting. BT, QY, ZC, and LX were involved in data collection and literature review. ZC did the manuscript revision. All authors have read and critically revised the manuscript for its intellectual content and approved the final version.

## Funding

This study was funded by Key Project of Chongqing Science and Technology Commission (cstc2018jscx-mszd0030), Personnel training project of National Regional Medical Center of Stomatological Hospital affiliated to Chongqing Medical University (QYYL2021A011), and Chongqing Medical University Future Medicine Youth Innovation Team development Support Plan – clinical innovation team (No.23).

## Conflict of Interest

The authors declare that the research was conducted in the absence of any commercial or financial relationships that could be construed as a potential conflict of interest.

## Publisher's Note

All claims expressed in this article are solely those of the authors and do not necessarily represent those of their affiliated organizations, or those of the publisher, the editors and the reviewers. Any product that may be evaluated in this article, or claim that may be made by its manufacturer, is not guaranteed or endorsed by the publisher.
