# Supplementary material for: Associations Between Oral Health Status, Perceived Stress, and Neuropsychiatric Symptoms Among Community Individuals With Alzheimer's Disease: A Mediation Analysis
Source: Front Aging Neurosci. 2022 Jan 10;13:801209. doi: 10.3389/fnagi.2021.801209 (PMC8786079; doi:10.3389/fnagi.2021.801209)
Supplement: Supplementary file 1 [file Data_Sheet_1.docx]

Supplementary Material

**1 Supplementary Tables**

Supplementary Table S1: Description of instrument used in the questionnaire survey

Supplementary Table S2: Top 10 oral microbiota based on relative abundance at phylum and genus level of SCD, MCI and AD groups

**Supplementary Table S1: Description of instrument used in the questionnaire survey**

| Scale | Description |
| --- | --- |
| MMSE^[1]^ | The Mini-Mental State Examination (MMSE) is a widely used standardized cognitive screening scale administered by trained clinicians and designed to detect cognitive impairment. It assesses cognitive domains, including seven aspects, a total of 19 items: orientation to time, orientation to place, registration, attention/calculation, recall, language (including naming, repetition, comprehension, reading, and writing), and copying. The total score ranges from 0 to 30, the higher score, the better cognitive function. A score of 24 or below suggests cognitive impairment. |
| CDR^[2]^ | The Clinical Dementia Rating scale (CDR) uses a semi-structured interview with both the patient and a reliable informant to assess the performance of cognitive functions in six domains: memory, orientation, judgment and problem solving, community affairs, home and hobbies, and personal care. Impairment is defined only when caused by cognitive loss rather than by physical disability or other non-cognitive factors. The scale helps neurologists rate the severity of dementia and related disorders on a 5-point scale from 1 (mild) to 3 (severe) based on clinical interviews with an informant and the person with dementia. Areas assessed include memory, orientation, judgment, problem-solving, community affairs, and home and hobbies. |
| NPI-12^[3]^ | The Neuropsychiatric Inventory (NPI) is designed be a self-administered questionnaire and widely used to evaluate neuropsychiatric symptoms (NPS). It was completed by interviewing subjects or their caregivers. NPI contained 12 symptoms: delusions, hallucinations, agitation, depression, anxiety, euphoria, apathy, disinhibition, irritability, abnormal motor behaviors, night-time behavior disturbances, appetite and eating disturbances. First, ask the patient whether there are related symptoms in the past 1 month. If there are no symptoms, it will be scored as 0. If there are, then further answer the subsequent questions and evaluate the frequency, severity of the symptoms and the degree of distress of the caregiver. Each of the 12 items is rated on a 4-point frequency scale(0 to 4) and a 3-point severity scale (0 to 3). The associated impact on the caregiver of the symptom manifestations (ie, Caregiver Distress) using a 5-point scale. The total score of each symptom was computed by product of frequency and severity score, ranging from 0 to 12 points. NPI scored from 0 to 144 points and higher score indicated more severe BPSD. |
| PSS^[4]^ | Perceived Stress Scale-10 (PSS-10) was usually used to assess the subjective experience of patients’ stress. It is a self-report questionnaire that assessed perceived stress over the past month. The scale included 6 negative items and 4 positive items which rated on a 5-point Likert scale ranging from “never” to “always” (0=Never, 1=hardly, 2=sometimes, 3=often, 4=always.). The total score (0 to 40) was computed by reversing positive items’ scores and summing up all items’ scores. A higher score indicates a greater perception of stress. |
| GOHAI^[5]^ | Geriatric Oral Health Assessment Index (GOHAI) was developed for assessing the self-reported oral health status in older adults. It intended to evaluate three different aspects of oral health-related quality of life, with 12 items: (1) physical functioning (4 items), including eating, speech, and swallowing; (2) psychosocial functioning (5 items), including worry or concern about oral health, dissatisfaction with appearance, self-consciousness about oral health, and avoidance of social contacts because of oral problems; and (3) pain or discomfort (3 items), including the use of medication to relieve pain or discomfort from the mouth. It was rated on a five-point scale ranging from “always” to “never” (1=always, 2=often, 3=sometimes, 4=seldom, 5=never). The total score was computed by reversing 3 negative items’ scores (always=5, never=1) and summing up all items’ scores, ranging from 12 to 60 points. A higher score indicated a better perceived oral health status and quality of life. |
| Oral health behavior^[6]^ | The Oral Health Behavior Questionnaire was developed by Ye et al.^[6]^ to assess oral health behavior of older adults in the community. The questionnaire contained two dimensions, oral hygiene behavior and oral condition handling behavior, with a total of 12 items. It scored using Likert 5-point ranging from 1 (never) to 5 (always). The total score is from 12 to 60 points, the higher the score, the better the oral health behavior. |

**Reference:**

[1] Folstein MF, Folstein SE, McHugh PR. "Mini-mental state". A practical method for grading the cognitive state of patients for the clinician. J Psychiatr Res. 1975,12(3):189-98. doi: 10.1016/0022-3956(75)90026-6.

[2] Morris JC. Clinical dementia rating: a reliable and valid diagnostic and staging measure for dementia of the Alzheimer type. Int Psychogeriatr. 1997;9(S1):173–176. doi:10.1017/S1041610297004870

[3] Leung VPY, Lam LCW, H F KC, et al. Validation study of the Chinese version of the neuropsychiatric inventory (CNPI). Int J Geriatr Psychiatry. 2001;16(8):789–793. doi:10.1002/gps.427

[4] Cohen S, Kamarck T, Mermelstein R. A global measure of perceived stress. J Health Soc Behav. 1983, 24(4):385-96. doi:10.2307/2136404

[5] Wong MC, Liu JK, Lo EC. Translation and validation of the Chinese version of GOHAI. J Public Health Dent. 2002 Spring;62(2):78-83. doi: 10.1111/j.1752-7325.2002.tb03426.x

[6] Ye S, Chen LQ. Development and validation of oral health knowledge belief and behavior questionnaire for community elderly. J Nurs Sci,2018,33(07):84-87. doi:10.3870/j.issn.1001-4152.2018.07.084

**Supplementary Table S2: Top 10 oral microbiota based on relative abundance at phylum and genus level of SCD, MCI and AD groups**

| Oral microbiota | SCD(n=32)  Mean(%) | MCI (n=32)  Mean(%) | AD(n=32)  Mean(%) | *P* | *Post-hoc* |
| --- | --- | --- | --- | --- | --- |
| p_Firmicutes | 31.49 | 37.57 | 39.84 | 0.04 | AD>SCD, MCI>SCD |
| p_Proteobacteria | 33.39 | 27.61 | 29.14 | 0.41 | n.s. |
| p_Bacteroidetes | 14.66 | 13.67 | 10.74 | 0.06 | n.s. |
| p_Fusobacteria | 9.21 | 10.58 | 7.67 | 0.17 | n.s. |
| p_Actinobacteria | 3.97 | 6.08 | 9.74 | 0.01 | AD>SCD, MCI>SCD |
| p_Patescibacteria | 3.30 | 1.53 | 1.27 | 0.00 | AD<SCD, MCI<SCD |
| p_Epsilonbacteraeota | 1.12 | 1.10 | 1.14 | 0.17 | n.s. |
| p_Spirochaetes | 1.91 | 1.06 | 0.16 | 0 | AD<SCD, AD<MCI |
| p_Synergistetes | 0.35 | 0.20 | 0.06 | 0 | AD<MCI<SCD |
| p_Cyanobacteria | 0.04 | 0.41 | 0.13 | 0.53 | n.s. |
| g_Burkholderia-Caballeronia- Paraburkholderia | 17.89 | 8.70 | 11.44 | 0.07 | n.s. |
| g_Streptococcus | 13.52 | 17.90 | 19.03 | 0.08 | n.s. |
| g_Neisseria | 7.87 | 10.69 | 10.49 | 0.93 | n.s. |
| g_Leptotrichia | 5.79 | 6.50 | 4.83 | 0.35 | n.s. |
| g_Veillonella | 3.86 | 4.64 | 6.94 | 0.11 | n.s. |
| g_Porphyromonas | 3.78 | 3.01 | 1.84 | 0.01 | AD<SCD, AD<MCI |
| g_Fusobacterium | 3.18 | 3.86 | 2.82 | 0.31 | n.s. |
| g_Prevotella_7 | 3.02 | 4.17 | 3.41 | 0.35 | n.s. |
| g_Capnocytophaga | 2.21 | 1.11 | 1.80 | 0.07 | n.s. |
| g_Prevotella | 2.14 | 2.00 | 1.10 | 0.01 | AD<SCD, AD<MCI |

Note: p, phylum; g, genus; n.s. no significant. Kruskal-Wallis test was used between three groups. The value of each microbiota was relative abundance.
